# Thriving in scrubs: a qualitative study of resident resilience

**DOI:** 10.1186/s12978-018-0489-4

**Published:** 2018-03-27

**Authors:** Abigail Ford Winkel, Anne West Honart, Annie Robinson, Aubrie-Ann Jones, Allison Squires

**Affiliations:** 1 New York University Langone Health, Department of Obstetrics & Gynecology, New York, NY USA; 2 New York University Rory Meyers College of Nursing, New York, NY USA

## Abstract

**Background:**

Physician well-being impacts both doctors and patients. In light of high rates of physician burnout, enhancing resilience is a priority. To inform effective interventions, educators need to understand how resilience develops during residency.

**Methods:**

A qualitative study using grounded theory examined the lived experience of resilience in residents. A cohort of obstetrics and gynecology residents were selected as a purposive, intensity sample.. Eighteen residents in all years of training participated in semi-structured interviews. A three-phase process of open coding, analytic coding and thematic analysis generated a conceptual model for resilience among residents.

**Results:**

Resilience among residents emerged as rooted in the resident’s calling to the work of medicine. Drive to overcome obstacles arose from personal identity and aspiration to professional ideals. Adversity caused residents to examine and cultivate coping mechanisms. Personal connections to peers and mentors as well as to patients and the work helped buffer the stress and conflicts that present. Resilience in this context is a developmental phenomenon that grows through engagement with uncertainty and adversity.

**Conclusion:**

Resilience in residents is rooted in personal and professional identity, and requires engagement with adversity to develop. Connections within the medical community, finding personal fulfillment in the work, and developing self-care practices enhance resilience.

## Plain English summary

Physician burnout occurs when doctors become cynical and hopeless in their work. Burned-out doctors may leave the workforce or suffer mental illness, and their care of patients is impaired. Burnout is common in medicine, and rises during medical training. In obstetrics and gynecology, more than half of residents suffer from burnout. Challenging work environments, emotional stressors and physical demands may contribute to burnout. Nonetheless, some physicians demonstrate resilience. Understanding how residents thriving in obstetrics and gynecology training engage with their work could help us understand how resilience develops in this context. A qualitative analysis of interviews with residents in obstetrics in gynecology led to a theory of resident resilience. In this context, resilience is tied to strong personal and professional identity. Resilience grows as residents deal with challenges during training, developing coping mechanisms, finding support in their colleagues and finding an authentic path as an individual doctor. This knowledge may help inform decisions around educational programs aimed at enhancing trainee wellbeing.

## Background

Physician burnout is rising and negatively affects physician and patient health [1–4]. Burnout increases throughout medical training [3, 5–14]. Long work hours, sleep deprivation, and challenging working environments influence burnout [6, 15–17]. Interventions to relieve burnout range from mindfulness training, reflective practice groups and cognitive behavioral therapy, to yoga classes and nutrition counseling to improve physician well-being [18–31]. While many of these seem to have a positive impact, the comparative effectiveness of these interventions is not clear [32–43].

*Resilience* is defined as “thriving despite adversity” [44]. While a resilient person may be easily recognized, researchers argue whether resilience is a fixed personal trait or a skill that can be learned [19, 24, 45, 46]. Internal factors such as personality traits, optimism and altruism may contribute [45, 47, 48]. In the face of adversity, behaviors including mindfulness and reflective practice may support a sense of control [47, 49–51]. External factors like social supports and healthy sleep are also protective in challenging situations [47, 50]. This suggests that resilience is both trait *and* state, and may be influenced by an individual and his or her surroundings. However, is not clear which of these factors are most relevant to physicians in training.

Medical trainees face unique stressors based on the content and context of their work. A conceptual model for resilience in medical students portrays the coping reservoir as a fuel tank [52]. Individual characteristics inform the architecture of the tank, and the level of fuel (or likelihood of displaying burnout) varies based on positive inputs or negative outputs. Once these students enter the workplace as doctors, it is not clear what the inputs are, or whether the model describes how resilience manifests.

A deeper understanding of resilience in physicians in training could focus effective interventions to improve well-being. Obstetrician-gynecologists encounter unique pressures and have high rates of burnout [5, 53]. This study examines the experiences of obstetrics and gynecology residents to generate a theory of how residents learn to thrive in this context.

## Methods

This was a prospective qualitative study using a constructivist approach and grounded theory methodology [54, 55]. Our research team consisted of a residency program director, two narrative medicine instructors, a resident and a PhD educator with expertise in qualitative methods. The research took place at New York University (NYU) Langone Medical Center, a large urban academic residency training program. The NYU Institutional Research Board (s16–01648) approved the study.

A theoretical, purposive sampling approach informed the selection of residents in good standing in obstetrics and gynecology training, whom we assumed to have an intense but not extreme experience of the phenomenon of resilience [55, 56]. The residency director provided a list of eligible participants to the research assistants, who contacted them individually. Recruitment continued until thematic saturation [57]; 18 residents (5 PGY-1, 6 PGY-2, 3 PGY-3 and 4 PGY-4) were included.

A semi-structured interview guide focused on concepts identified by prior research as relevant to resilience in other contexts [58]. After consenting to participate, residents gave semi-structured interviews lasting 45–60 min. Research assistants who knew the residents but who were not involved in clinical training or evaluation performed the interviews. Digital recordings of the interview were professionally transcribed verbatim and then de-identified. The program director and resident members of the research team did not have access to any de-identified transcripts or data.

A systematic grounded theory analysis employed three phases of coding [59]. First, two coders assigned inductive, content-driven labels to text segments. Serial discussions resulted in homogenized codes and a uniform codebook stabilized after nine interviews to include 54 codes [60]. Reflective memos tracked insights related to the data [55, 56]. Using constant comparison, analytic coding developed major categories around the core construct of “being a doctor” (Table 1). The thematic coding phase defined the relationships between categories as themes, which explained a conceptual model. After 18 interviews, the researchers recognized thematic saturation as they understood not only the concepts raised by the participants, but also the meanings attributed to them [57]. To enhance credibility, reflexive memos, interview notes, and member checking with participants explored the subjectivity within the analysis [61].

**Table 1:** Categories and Code Members

Major Categories	
Being a Doctor	Central construct; includes thoughts about professional identity, passion for the work, and navigating the boundaries of personal versus professional life.
Background	Family, influential early experiences, genesis of decisions to pursue career path
Values	Perceptions and expectations of self and others, emphasis on altruism/pay it forward, responsibility, perfectionism, compassion. Reflections on spirituality and religion.
Fuel	What drives you? Persistence, self-directedness, planning, positive attitude. Navigating challenges through looking forward to the future as well as backwards with pride in growth. Faith in the universe, reflections on control.
Support System	Relationships within and without medicine support from family and significant other. Importance of teamwork and mentors, especially within the residency group itself.
Attention to Self	Self-awareness, coping strategies, self-care. Reflection and meaning. Identity within medicine and as “normal people”.
Tensions	Values conflicts, difficult clinical material in medicine, tensions within teams and other adversity. Death, end of life, burnout and strategies to attend to mental health.

## Results

### Navigating personal and professional identity

The concept of “becoming a doctor” emerges throughout the interviews as the core construct within professional resilience. Residents shoulder the frustrations and challenges of the work as they reach towards high ideals for the profession and high expectations for themselves. When the challenges are overwhelming or the ideals feel particularly far away, they describe feelings of burnout. They find solidarity and consolation through connection with others in the medical community, especially with fellow residents. As one resident puts it, “medicine is a team sport. Everyone here has the same end goals as residents to offer the best care to our patient that we can” (PGY-3, Subject 9). The other residents are “really the only people who understand what’s going on in a very real way.” (PGY-4, Subject 10). Residents frequently used war metaphors and describe each other as being in the army together. But not all residents bond with the others, and one struggling resident described not feeling support from this team spirit, saying, “you’re constantly working with one another. Everything feels like a group project. I’ve always hated that.” (PGY-2, Subject 20).

Residents frequently express stress and frustration when there are conflicts within the resident team or with allied health professionals. Even among residents, competitiveness and hierarchy simmer beneath the surface. Some residents, more commonly junior residents expressed less comfort exposing their vulnerabilities within the medical community. One says,

*There’s a tendency in medicine to not show weakness and I think that that’s very hard to overcome. There are like a couple of people who I would feel comfortable talking to about really feeling bad or if I have very heavy things on my chest, but not everybody. (PGY-2, Subject 21).*

In addition to hierarchy and competition, which coexist in the resident team, the residents also personally rely on each other to cover the clinical services. When one resident’s personal needs cause an absence, others must work harder.

Thus medical relationships, while providing a major source of support to residents, nonetheless reflect a surrounding culture that espouses herculean ideals. Many residents look to their relationships outside of medicine to provide more balance and accommodation for their own needs and imperfections. The tension between professional and personal identities reveals itself as residents talk about “normal people” who do not work in healthcare. The residents both pride themselves in having a distinct professional identity, and also envy these others. From the residents’ perspective, normal people are permitted to focus on their personal needs, while doctors feel guilt when attending to theirs.

Families and friends provide strong support to the residents, and are seen as a counterbalance to the professional world. One resident describes speaking to her boyfriend, saying:

*When I’m like, ‘oh, this is such a terrible thing, this day was crazy,’ he’s like, ‘but you’re fine and you’re here and everything seems like it’s okay.’ And then you’re like, ‘yes, in the grand scheme of things not faxing that slip in for the [surgical] instruments was totally fine.’ (PGY-3, Subject 4).*

These perspectives from outside the medical world provide useful counterbalance to germane work stresses, but residents also report keeping some of the more traumatizing experiences private. One describes not wanting to burden her partner with “the sad things that we see and are adapted to” (PGY-2, Subject 22). Building relationships and families during this stage of adulthood is developmentally important, but can be challenging to balance with work. As one reflects, “it’s been hard to be an OB/GYN and feel like everyone around you is having babies and moving forward in their lives and you’re at this place where residency doesn’t allow you to move forward in your personal life” (PGY-2, Subject 20). Residents place a lot of attention on finding crossroads between their personal and professional identities. When these efforts are successful, they are more likely to portray resilience.

### Calling to the profession

For many residents, the call to medicine comes from early life experiences, which inform their professional aspirations and identity. Residents describe parents who were doctors, or who prized hard work and high achievement. As one resident says:

*Wanting to do well and succeed was instilled by two successful parents who gave me a lot and wanted me to do well and succeed, wanting to make myself and my family proud. (PGY-2, Subject 22).*

This achievement-oriented approach to the work influences how they engage with obstacles.

In the face of difficult experiences, residents often reflect on their future goals or take pride in what they have learned through prior struggles. Persistence and a “positive attitude” are prized values. One resident remarks, “If you have a positive attitude, you’re enthusiastic, and willing to work hard, that can get you through pretty much anything” (PGY-4, Subject 8). The same resident says that “the hardest part of residency is you go from 20-plus years of being in school where you get a gold star, you get an A, you get these pats on the back.” In clinical work, where these rewards are not always visible, the residents can get discouraged. During residency, “this is just the expectation, this is what you’re doing, this is a job’ and there’s no one to be like, ‘wow, that was a great job you did today.” (PGY-4, Subject 8).

Some residents identify moments where they discover private rewards in their connections to patients. One says,

*My favorite, which happens when nobody’s looking, is when the patient says, ‘Can I come back and see you? You’re so good. Thank you so much. That was the first time somebody’s explained this to me.’ (PGY-2, Subject 6)*.

Reframing these connections as the rewards of their efforts requires focusing on the patient and not their own aspirations. Some residents describe other-motivated motivations in the form of values of duty, responsibility or altruism. Often this appeared in a motivation to work with underserved populations. Taking a step back to see the value of the connection to the patient provides another personal support for the work. One resident says, “what is more intimate that being in the room and delivering somebody’s baby…we appreciate how intimate that moment is, and how special it is to get to be a part of it. (PGY-3, Subject 9) This process of finding something special and important within the work itself and the patients, rather than looking for evidence of the doctor’s achievement seemed to provide a way to stay engaged with the professional calling despite struggles endemic in the work, and without obvious external rewards for achievement.

### Connection between self-care and empathy

Residents make connections between their own wellbeing and their ability to connect with patients. When they are not feeling cared for themselves, it is harder for them to feel empathy for patients. One resident says she lost her empathy when feeling burned out. She says her peers, “who are further on down the line say that it comes back, when you’re more rested” (PGY-2, Subject 20). There is a perceived conflict between being the caregiver and caring for themselves. As one says, “we all give out great advice to each other but we don’t always take it ourselves” (PGY-2, Subject 5). Reflections on self-care often focus on basic needs of food and rest, attention to nurturing the physical self. Many recognized intense levels of stress around them, but feel there is little room reflection and processing the stressful and traumatic aspects of training. As one states, “after a bad outcome, there is a little bit of repression because you have to run to the next thing. There’s not a lot of time to process in the moment.” (PGY-2, Subject 21) This kind of short-term compartmentalization is widely endorsed as necessary to function in the fast-paced and demanding clinical environment. A PGY-4 resident recalls, “no, I cannot do this right now. I need to come back to this because I'm not thinking clearly about it. I will come back to this and I will deal with it” (PGY-4, Subject 10). Later, if the incidents are unpacked, the residents sometimes uncover unwanted self-critical and perfectionist thoughts. One resident says, “I tend to tell myself that, no, that was horrible, you know, you could have done this better, you could have done that better” (PGY-1, Subject 2).

Residents see both negative and positive role models among senior residents and faculty, and many recognize that cultivating effective self-care and coping mechanisms are important to avoiding cynicism. Surprisingly, despite stigma and a medical culture that discourages showing weakness, reflections on mental health services were common and largely positive. Residents display comfort with having sought and benefited from mental health treatments. The immediate requirement to prioritize patient before self in a complicated situation requires an effective means of processing the experience later. For some, they do not revisit these experiences for themselves, and the protective boundaries build up, distancing the doctor from the patient. Loss of empathy threatens the patient but also removes an important source of motivation – that connection – from the doctor.

### Learning through adversity and uncertainty

Resilience emerges as the residents engaged with challenges. For those who developed resilience through difficult experiences prior to residency, the trials of residency: struggling with self-doubt, the weight of responsibility and making mistakes, appear less overwhelming. For many in this group, control and persistence characterize their approach to problems. But some of the adversity and uncertainty experienced in residency does not present a problem which can be solved. When patients suffering cannot be explained, when complications cannot be reversed, when treatments are not effective, these experiences shake their conviction in the profession.

In the face of daunting challenges and difficult clinical encounters, many describe distance from their spiritual base, even if they endorsed a prior religious or spiritual orientation. One resident describes the way this has eroded during training, saying:

*I think a lot of people would like to believe that there’s something out there that’s more than themselves. I feel like working in healthcare and seeing all the bad things that happen to people makes that hard. I don’t know if you can see [these things] over and over and over again and think, ‘oh yeah, this is definitely the plan.’ (PGY-1, Subject 1).*

For many members of this group, residency is the first point in their lives when things did not follow their own “plan”. Learning to tolerate these experiences and find other ways of managing stress beyond control and persistence is a big part of the path to resilience for many of these residents.

### Conceptual model of resident resilience

**Fig. 1:**
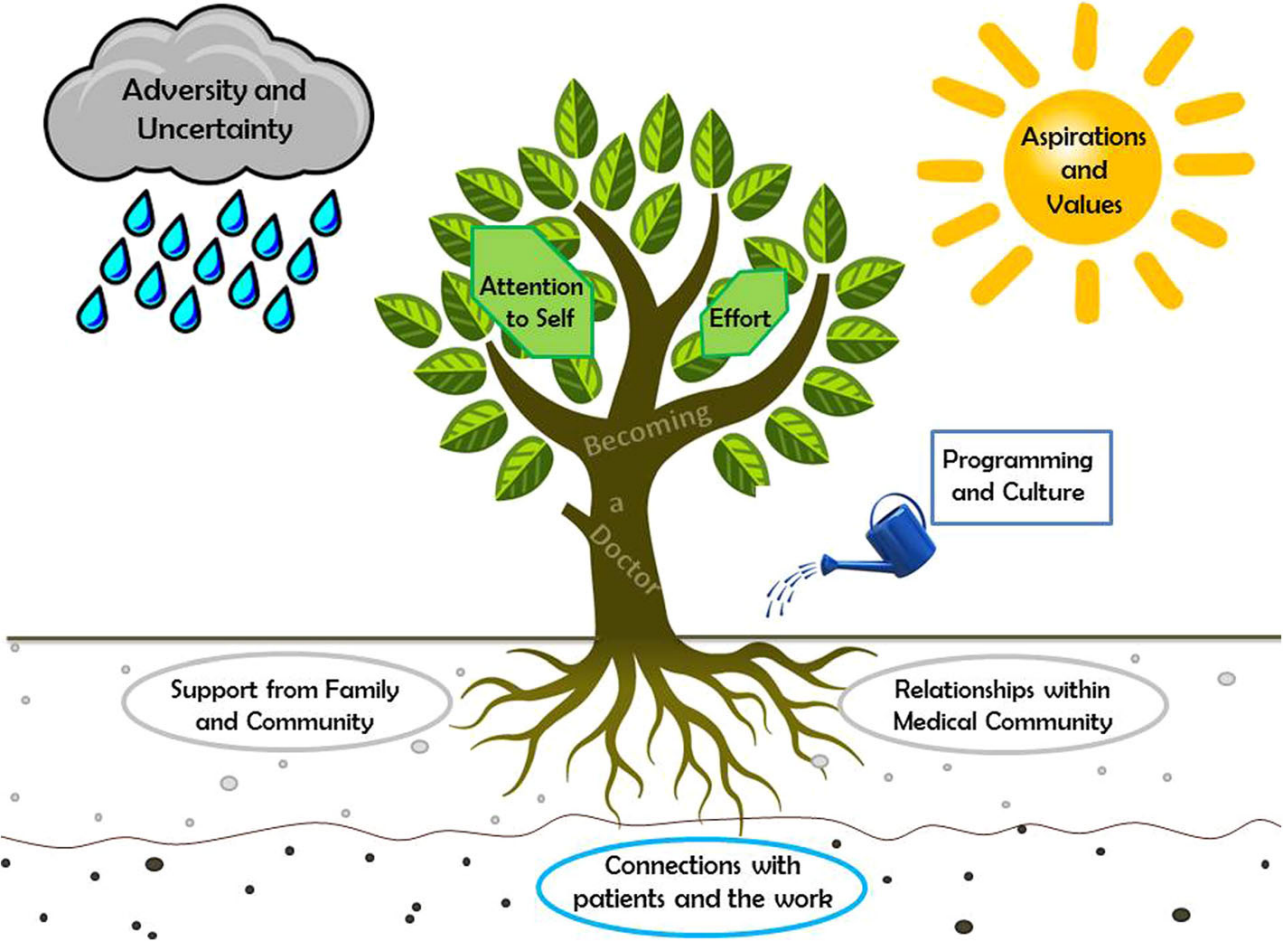
Conceptual Model of Resilience in Residents. Resilience emerged as a developmental phenomenon, rooted in the resident’s family and community values. As the physician grows into a doctor, the root bed expands to peers and mentors within the medical community, and branches stretch in the direction of aspirational values. Uncertainty and adversity provide challenges but also fuel growth and define professional identity

These concepts generate a dynamic and developmental model of resilience in obstetrics & gynecology residents that resembles a growing organism. Represented here by a tree, (Fig. 1) the core of resilience is the process of becoming a doctor. The roots emanate from family and community values, and the tree reaches upwards towards archetypal professional values and aspirations, represented by the sun. The root bed expands to connections within the medical community, and deepens through connections to patients and the work. Storms of adversity, uncertainty, challenges and value conflicts blow through and shake the tree, but also provide water that allows the tree to grow. Leaves represent the fuel for growth, both the sheer determination embodied by this group as well as development of coping mechanisms which expand the capacity to grow. A watering can demonstrates the ways that programmatic interventions and positive culture might further cultivate the evolution of this growing entity.

## Discussion

This analysis generates a model for resilience as a phenomenon that emerges as physicians in training engage with challenges and difficult experiences. Motivation to persist comes from an initial desire to live up to ideals of the profession, supported by the resident’s personal community, and by socialization into the profession. Resilience manifests as the ability to re-engage with the work when threats arise to these ideals. Developing resilience requires that residents attend to their own individual humanity, and find personal connections to patients and the work.

This model of resilience in OBGYN residents contains elements recognized as important to resilience in other populations. These include optimism, strong value systems, social support and role models, embracing physical and mental challenges, and finding meaning in challenges [62]. Conspicuously absent are other factors that emerge in resilient populations outside of this context such as drawing on faith for support, embracing cognitive and emotional flexibility and facing fears. The adversity that doctors face differs from the kind of traumas experienced by these other populations who displayed resilience despite violence, death or disaster. The chronic and undulating nature of stresses in medicine are likely different, and the boundaries between personal and professional lives may influence what is needed for resilience to develop within the work. Learning through examining professional mistakes and difficult outcomes is emotionally threatening, and the medical culture may not naturally nurture resilience.

Unlike the fixed, “gas-tank” model for resilience in medical students, [52] resilience in residents emerged as an organic organism, forming itself through the transformative process of residency. This has implications for understanding what might help cultivate resilience among trainees. Unlike a coping reservoir tank which might simply be filled by a positive input, adaptive coping must be developed as a process of dealing with ongoing threats and changes as the doctor grows in the work.

The strength of this research is that it provides a rich description of how resilience develops among a group of trainees at different levels of training working in a physically, intellectually and emotionally challenging environment. By seeing not only whether resilience is present or not, but rather coming to understand the process of developing resilience, insights from the study may help inform choices about programming that create a culture that facilitates this process.

Limitations to the research within the study design include the choice of questions within the study guide, which relied on prior research on resilience and may therefore have missed or stressed particular elements. The background of the research team in narrative medicine may have informed the approach to interviews and analysis. The narrative medicine approach encourages physicians to reflect on their relationships to patients, to themselves, and to society [63]. Thus, ideas related to professional identity, empathy and humanism might have emerged more prominently.

Resilience has been measured by a heterogeneous group of instruments, each measuring different aptitudes [46, 48, 50, 64, 65]. We did not include an objective measure of resilience as part of the study design, and from the interviews, it seemed that despite apparent thriving, many of the participants had experienced burnout. Nevertheless, the model of resilience generated by the interviews suggested that this phenomenon is a process rather than a fixed state or trait, and these individuals might be resilient or not-resilient depending on the particular circumstances.

Understanding resilience based on this model has some implications for educators. The heavy emphasis on identity and learning through challenges suggests that curricula using reflection to explore experiences might help cultivate resilience. Programmatic interventions (e.g. Narrative Medicine workshops [66, 67] or Balint groups [68] or Schwartz Rounds [69]) may have the potential to encourage engagement with the work. The importance of team and community to facilitate or inhibit the development of resilience suggests that attention be paid to support professional relationships with colleagues and mentors. Future research is needed to determine whether programmatic changes based on these insights about the nature of resilience result in improved wellbeing among trainees.

## Conclusions

Resilience in the context of obstetrics & gynecology residency training emerges through this grounded theory inquiry as a developmental phenomenon grounded in the physicians calling. Through finding support from colleagues and connecting with meaning in the work, the initial call to the work of caring for patients is strengthened rather than weakened by difficult experience. Building this capacity for resilience requires expansion of coping mechanisms and reflection on the uncertainty and adversity presented by the training experience. Understanding resilience in this way may help inform the efforts of educators who aim to enhance resilience through program structure and curricula.
